# Sublethal Paraquat Confers Multidrug Tolerance in *Pseudomonas aeruginosa* by Inducing Superoxide Dismutase Activity and Lowering Envelope Permeability

**DOI:** 10.3389/fmicb.2020.576708

**Published:** 2020-09-25

**Authors:** Dorival Martins, Geoffrey A. McKay, Ann M. English, Dao Nguyen

**Affiliations:** ^1^Meakins-Christie Laboratories, Research Institute of the McGill University Health Centre, Montreal, QC, Canada; ^2^Department of Microbiology and Immunology, McGill University, Montreal, QC, Canada; ^3^Department of Chemistry and Biochemistry, Concordia University, Montreal, QC, Canada; ^4^Department of Medicine, McGill University, Montreal, QC, Canada

**Keywords:** *Pseudomonas aeruginosa*, antibiotic tolerance, stationary phase, superoxide dismutase, superoxide generators, paraquat, stringent response, RpoS

## Abstract

Stressors and environmental cues shape the physiological state of bacteria, and thus how they subsequently respond to antibiotic toxicity. To understand how superoxide stress can modulate survival to bactericidal antibiotics, we examined the effect of intracellular superoxide generators, paraquat and menadione, on stationary-phase antibiotic tolerance of the opportunistic pathogen, *Pseudomonas aeruginosa*. We tested how pre-challenge with sublethal paraquat and menadione alters the tolerance to ofloxacin and meropenem in wild-type *P. aeruginosa* and mutants lacking superoxide dismutase (SOD) activity (*sodAB*), the paraquat responsive regulator *soxR*, (p)ppGpp signaling (*relA spoT* mutant), or the alternative sigma factor *rpoS*. We confirmed that loss of SOD activity impairs ofloxacin and meropenem tolerance in stationary phase cells, and found that sublethal superoxide generators induce drug tolerance by stimulating SOD activity. This response is rapid, requires *de novo* protein synthesis, and is RpoS-dependent but does not require (p)ppGpp signaling nor SoxR. We further showed that pre-challenge with sublethal paraquat induces a SOD-dependent reduction in cell-envelope permeability and ofloxacin penetration. Our results highlight a novel mechanism of hormetic protection by superoxide generators, which may have important implications for stress-induced antibiotic tolerance in *P. aeruginosa* cells.

## Introduction

Bacteria can survive the lethal effects of antibiotics through expression of genetically inheritable resistance mechanisms. They also can adopt a transient physiological state of drug tolerance (Levin and Rozen, 2006; Meylan et al., 2018), which is widely observed in slow growing and biofilm bacteria. Such a drug tolerant state likely contributes to chronic infections refractory to antibiotic treatment, particularly those caused by the major human opportunistic pathogen *Pseudomonas aeruginosa*.

A wide range of physiological, metabolic and environmental stressors can induce drug tolerance and lower antibiotic lethality. For example, nutrient starvation (Nguyen et al., 2011; Bernier et al., 2013), ATP depletion (Conlon et al., 2016), respiratory inhibition (Lobritz et al., 2015), transition to stationary phase (Davey et al., 1988) and hypoxic environments (Walters et al., 2003; Stewart et al., 2015) dampen antibiotic killing, while nutrient utilization that enhance TCA cycle activity and aerobic respiration enhance drug lethality (Allison et al., 2011; Dwyer et al., 2014; Meylan et al., 2017). Our groups and others have previously shown that global stress responses (Chen et al., 2009; Lewis, 2010; Harms et al., 2016), such as those mediated by the alternative sigma factor RpoS (Murakami et al., 2005) and (p)ppGpp signaling (Nguyen et al., 2011), confer multidrug tolerance in *P. aeruginosa.* As such, the mechanisms of drug tolerance are likely multifactorial, condition specific, and species-specific.

How oxidative stress pathways contribute to bacterial survival when challenged with bactericidal antibiotics remains a complex and incompletely understood question. Superoxide radicals can contribute to cell death by inactivating iron-containing proteins, particularly those harboring [Fe-S] clusters that release Fe ^2+^ to catalyze the production of highly reactive hydroxyl radicals by Fenton chemistry (Hausladen and Fridovich, 1994; Keyer and Imlay, 1996). While several groups have previously reported that bactericidal antibiotics induce production of reactive oxygen species, including superoxide and hydroxyl radicals, which contribute to their off-target killing mechanism (Dwyer et al., 2007; Grant et al., 2012; Sampson et al., 2012; Imlay, 2013; Van Acker et al., 2016), others have refuted these observations (Keren et al., 2013; Liu and Imlay, 2013). Superoxide stress also induces anti-oxidant defenses such as superoxide dismutases (SOD), which in turn modulate antibiotic lethality as we and others have reported (Bizzini et al., 2009; Hwang et al., 2013; Ladjouzi et al., 2013, 2015; Heindorf et al., 2014; Wang et al., 2014; Martins et al., 2018). We recently demonstrated that induction of SOD activity confers multidrug tolerance to stationary phase *P. aeruginosa* through alteration of the cell envelope permeability and increased drug accumulation (Martins et al., 2018).

Superoxide-generating compounds, such as paraquat (PQ), menadione (MN) and plumbagin, increase intracellular superoxide levels. Although they can cause superoxide mediated damage, and are thus expected to amplify cell death, they have also been reported to reduce antibiotic susceptibility and mitigate killing in some studies (Wu et al., 2012; Mosel et al., 2013). Superoxide-generating compounds induce gene expression through activation of the transcription factors SoxR, and OxyR (Ochsner et al., 2000; Blanchard et al., 2007), including genes encoding drug efflux systems in *Escherichia coli*, *P. aeruginosa*, and other species (Miller et al., 1994; Pérez et al., 2012; Mosel et al., 2013; Blanco et al., 2017). However, how superoxide stress alters the susceptibility of *P. aeruginosa* to antibiotic lethality remains poorly understood. In this study, we report that sublethal PQ and MN confer antibiotic tolerance in stationary phase *P. aeruginosa* cells by inducing SOD activity. This SOD response is rapid, RpoS-dependent but (p)ppGpp- and SoxR-independent, leading to a reduction in envelope permeability and drug accumulation, and diminished killing by ofloxacin (a quinolone) and meropenem (a beta-lactam) in stationary phase cells.

## Materials and Methods

### Bacterial Strains and Culture Conditions

The bacterial strains used in this study are listed in Table 1. All *Pseudomonas aeruginosa* mutants are derived from the parental wild-type (WT) strain PAO1. The Δ*soxR* mutant harboring an unmarked *soxR* deletion was constructed by allelic exchange using the plasmid pSMV10-Δ*soxR* (Dietrich et al., 2006). Merodiploids were selected for gentamicin resistance, followed by counterselection on 15% sucrose and confirmation of the mutation by PCR and sequencing. The *sodAB* mutant was generated by homologous recombination of the *sodB* mutation into a *sodA* mutant using genomic DNA from the *sodB* mutant (Iiyama et al., 2007), and selection with 90 μg/mL tetracycline and 75 μg/mL gentamicin.

**TABLE 1:** Bacterial strains.

**Strain name (ID)**	**Description**	**Source**
WT (DN276)	*Pseudomonas aeruginosa* PAO1 wild-type strain	Nguyen et al., 2011
*sodA* (DN914)	PAO1 *sodA* mutant *sodA:*Ω*aaC1*, Gm^r^	Iiyama et al., 2007
*sodB* (DN916)	PAO1 *sodB* mutant *sodB:*Ω*Tc*, Tc^r^	Iiyama et al., 2007
*sodAB* (DN1106)	PAO1 *sodA sodB* mutant *sodA:*Ω*aaC1*, Gm^*r*^ *sodB:*Ω*Tc*, Tc^r^	This study
Δ*relA*Δ*spoT* (DN23)	PAO1 Δ*relA*Δ*spoT* with Δ*relA* (Δ181-2019) Δ*spoT* (Δ200-1948) unmarked deletions	Nguyen et al., 2011
*rpoS* (DN705)	PAO1 *rpoS* transposon mutant with *rpoS-*B03: IS*lacZ/hah* allele from PW7151; Tc^r^	Held et al., 2012
Δ*soxR* (DN1105)	PAO1 Δ*soxR* mutant with Δ108–281 deletion in the *soxR* gene replaced by cccatccactaaatttaaata	Dietrich et al., 2006
WT + vc (MK318)	PAO1 (DN276) with vector control attTn7:miniTn7-Gm, Gm^r^	Khakimova et al., 2013
WT + *katA* (MK298)	PAO1 (DN276) with *pBAD-katA* construct miniTn7-Gm-GW*-araC-*pBAD*-katA* chromosomally inserted at the *attTn7* site, Gm^r^	Khakimova et al., 2013

*We note that the isogenic PAO1 parental strain to the rpoS and sodAB mutants originated from different sources than the WT (DN276) PAO1 strain. Control experiments with all PAO1 strains showed no differences in SOD activity, antibiotic tolerance or growth characteristics (data not shown).*

All bacterial cultures were grown in LB Miller liquid medium (wt/v 1% tryptone, 0.5% yeast extract and 1% NaCl, Difco) or 1.5% agar (wt/v). Single colonies grown overnight on LB agar plates from glycerol stocks were picked to inoculate starter cultures (5 mL LB in 25 mL slanted tubes), grown for 8 h, then sub-cultured to an initial OD_600_ = 0.05 and grown to exponential (2 h, OD_600_ = 0.2) or late-stationary phase (16 h) in 15 mL LB in 150 mL flasks. All liquid cultures were grown at 37°C with shaking at 250 rpm. In order to generate a robust SOD induction in response to sublethal PQ and MN challenge, stationary phase cultures were diluted 10-fold in their own spent medium (without new nutrients) to OD_600_ = 0.3, namely filter sterilized (0.22 μm filters) supernatants of the same stationary phase cultures, prior to PQ or MN challenge. To induce *katA* expression from cells transformed with the pBAD-*katA* construct, 2% (w/v) L-arabinose (Sigma, #A3256) was added. Gentamicin 75 μg/mL (Sigma #G1264) and tetracycline 50 μg/mL (Sigma #T6660) were used for selection where appropriate.

### Paraquat (PQ) and Menadione (MN) Challenge

After 16 h growth, stationary phase cells were diluted 10-fold (final OD_600_ ∼0.3) in their spent culture medium unless otherwise specified. Cells were transferred to 96-well plates and incubated with sublethal concentrations of the superoxide generators PQ (Sigma #856177) or MN (Sigma #M5625) at 37°C with shaking at 250 rpm. Unless otherwise specified, cells were challenged 1.25 mM PQ, 0.175 mM MN or an equivalent volume of the vehicle (MilliQ H_2_O for PQ or DMSO for MN) for 20 min before the SOD activity was assayed, or before antibiotics were added for the antibiotic killing assays.

### SOD and Catalase Activity Assay

Cells from ∼10 mL of diluted stationary phase cultures (OD_600_ = 0.3) pre-challenged with PQ, MN or vehicle control, were pelleted by centrifugation at 10,000 × g for 5 min at room temperature, washed twice with phosphate buffered saline (PBS), resuspended in 0.25 mL PBS (10 mM sodium phosphate, 150 mM NaCl, pH 7.4) and lysed by sonication on ice (100 W, 6 × 10 s cycles on ice). The lysates were spun at 12,000 × g for 5 min to remove cell debris; the supernatants containing soluble proteins were collected and assayed for total protein (Bio-Rad Bradford assay), SOD or catalase activity.

SOD activity was assayed as done previously (Martins et al., 2018). Briefly, 2.5–10 μL of soluble protein extracts (5–20 μg in the final assay) were added to a solution containing 50 mM potassium phosphate (KPi) buffer (pH 7.5), 30 μM horse heart ferricytochrome c (Sigma #C6749) and 100 μM xanthine (Sigma #X0626). The assay was started by the addition of 0.25 μg/mL xanthine oxidase (Sigma #X4875), and reduction of ferri- to ferrocytochrome c was measured via absorbance at 550 nm (Δε_550_ = 19.6 mM^–1^ cm^–1^) in a 96-well plate reader (Tecan Infinite M1000). One unit of specific SOD activity per mg of protein (U/mg) inhibits the rate of ferricytochrome c reduction by 50%. Catalase activity was assayed as done previously (Martins et al., 2018). Briefly, 25–150 μL soluble protein extract (0.5–10 μg in the final assay) was added to 1.0 mL of 20 mM H_2_O_2_ in 50 mM KPi and H_2_O_2_ decomposition was monitored via absorbance at 240 nm (ε_240_ = 43.6 M^–1^cm^–1^) (Beers and Sizer, 1952). One unit of catalase activity catalyzes the degradation of 1 μmol of H_2_O_2_ per min (Beers and Sizer, 1952; Martins and English, 2014).

### Antibiotic Killing Assays

Stationary phase cells were diluted 10-fold in their spent medium, pre-challenged with PQ or MN (where indicated) then transferred to 96-well plates (200 μL final volume) for challenge with 5 μg/mL ofloxacin (Sigma #O8757) or 500 μg/mL meropenem (Sigma #M2574) at 37°C with shaking at 250 rpm. At specific time points, 25 μL was removed, mixed with an equal volume of activated charcoal (25 mg/mL in PBS, Sigma #05105) to bind the residual antibiotic, serially diluted in sterile PBS and plated on LB agar plates for counting of colony forming units (CFU) after overnight growth at 37°C. The vehicles (50 μM aqueous NaOH for ofloxacin, MilliQ H_2_O for meropenem) were used as controls.

### Inhibition of *De Novo* Protein Synthesis

Stationary phase cells were diluted 10-fold in their spent medium and treated with 500 μg/mL chloramphenicol (∼10× the minimal inhibitory concentration) for 1 h to inhibit *de novo* protein synthesis. Cells were then challenged with 1.25 mM PQ for 20 min before harvest to prepare the soluble protein extracts for SOD activity assays. To validate the inhibition of *de novo* protein synthesis, WT cells expressing an arabinose-inducible *katA* construct (pBAD*-katA*) or a control vector were grown to stationary phase, diluted 10-fold in their own spent medium and incubated with ± 2% L-arabinose (wt/v) ± 500 μg/mL chloramphenicol. Soluble-protein extracts were prepared 1.25 h later for measurement of catalase activity. All incubations were done at 37°C with shaking at 250 rpm.

### Ethidium Bromide (EtBr) and Dihydroethidium (DHE) Staining

EtBr internalization is an indicator of envelope permeability (Ocaktan et al., 1997), whereas DHE staining reports on relative intracellular superoxide levels (Lee K. et al., 2009). Staining was carried out as described before with minor modifications (Martins et al., 2018). Where indicated, stationary phase cultures were pre-challenged with PQ or MN as described above, and without washing, cells were stained with 15 μM EtBr (Sigma #E7637) or 15 μM DHE (Thermo Fisher Scientific #D11347) for 1 h at room temperature in the dark without shaking. Since they are substrates for efflux pumps, EtBr and DHE staining was carried out in the presence of 100 μM carbonyl cyanide *m*-chlorophenyl hydrazine (CCCP, Sigma #C2759), a protonophore that inactivates H^+^-dependent efflux systems. Stained cells were fixed with 4% formalin (v/v) and analyzed by flow cytometry (BD Accuri C6 flow cytometer, BD Biosciences). Relative fluorescence units of individual bacterial cells were determined at Ex/Em 490/580 nm for EtBr and the DHE superoxide-reaction products (namely, 2-hydroxyethidium), and the median fluorescence intensity (MFI) of 10,000 cells was reported for each sample. To estimate superoxide levels, we calculated the MFI ratio of DHE/EtBr fluorescence in each sample to correct for probe loading (Martins et al., 2018).

### Efflux Pump Activity

The relative H^+^-dependent efflux activity was estimated from the ratio of EtBr MFI in the presence or absence of CCCP (+CCCP/-CCCP ratio) under the specified conditions. Stationary phase cells were stained with 15 μM EtBr with or without 100 μM CCCP for 1 h at room temperature in the dark without shaking. EtBr fluorescence was measured by flow cytometry as above.

### Ofloxacin Internalization Assay

Ofloxacin internalization was assessed by measuring intracellular drug levels in stationary phase WT cells. Briefly, stationary phase cells were diluted to OD_600_ 0.5 in their spent media, and pre-challenged with 1.25 mM paraquat or H_2_O (control) for 20 min, then incubated for 1 h with 0.5 μg/mL ofloxacin, a sublethal concentration chosen to cause no cell death during the incubation period. Cells were harvested by centrifugation, washed once in 10 mL PBS and resuspended in 250 μL ddH_2_O to which an equal volume of methanol was added to lyse cells and extract ofloxacin. Samples were vortexed vigorously for 2 min at room temperature and centrifuged twice (14,000 × g for 20 min) to remove cell debris. The supernatant was collected and stored at −20°C until analyzed.

Quantification of ofloxacin was performed by liquid chromatography-mass spectrometry (LC/MS) using multiple reaction monitoring in positive mode on a triple quadrupole MS system (EVOQ Elite, Bruker) coupled with an ultrahigh-performance liquid chromatography (LC) pump (Advance, Bruker) and a reversed-phase Agilent Zorbax Eclipse Plus C18 column (2.1 × 50 mm, 1.8 μm; P.N. 959757-902). Mobile phases were water with 0.1% formic acid (A) and acetonitrile with 0.1% formic acid (B). 10 μL samples were injected and ofloxacin was eluted at 2.53 min with the following LC conditions: 0–1 min at 5% B, from 1–6 min with gradient up to 95% B, followed by a plateau at 95% for 2 min, and back to 5% B at 8.1 min until 10 min, with a column temperature set at 40°C and flow rate at 0.4 mL/min. Two mass transitions were followed for ofloxacin 362.0 → 318.0 (CE 17 eV), 362.0 → 260.0 (CE 25 eV). The lower limit of detection (LOD 5 pg/mL) and calibration curve (range 15.5–1,000 pg/mL were established using ofloxacin standards (>99%; Sigma-Aldrich #O8757). The relative ofloxacin concentration was calculated as area under the curve (AUC) and the mean of three technical replicates was reported for each sample.

### Statistical Analyses

Results were pooled from at least two independent experiments, each performed with at least three biological replicates as indicated. Two-tailed Student’s *t*-test was used to compare two conditions and one-way analysis of variance (ANOVA) with Tukey multi-comparison post-test was used for comparison between three or more conditions. The correlations between SOD activity and survival to antibiotic challenge were established using non-linear regression (second order polynomial). *P* ≤ 0.05 were considered statistically significant. Statistical analyses were done using the Prism 7 software (GraphPad, CA, United States).

## Results

### The *sodAB* Mutant Is Highly Impaired for Antibiotic Tolerance in Stationary Phase *P. aeruginosa*

**FIGURE 1:**
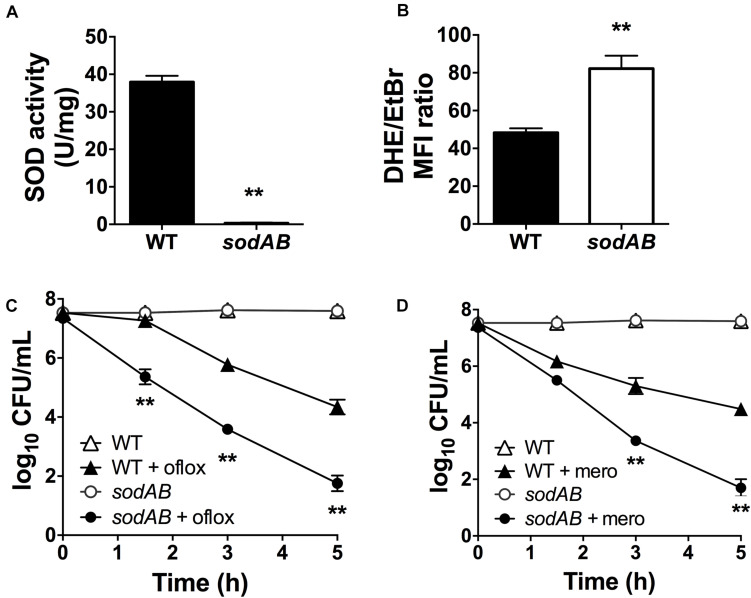
Loss of SOD activity impairs drug tolerance in stationary-phase *Pseudomonas aeruginosa.* **(A)** SOD activity, **(B)** relative intracellular superoxide levels and tolerance to killing with **(C)** 5 μg/mL ofloxacin or **(D)** 500 μg/mL meropenem in wild-type (WT) and *sodAB* cells. Relative intracellular superoxide levels were calculated from the ratio of DHE/EtBr median fluorescence, both measured in the presence of 100 μM CCCP. Note that the data points for WT and *sodAB* cells without antibiotics overlap in **(C,D)**. Results are shown as mean ± SEM (*n* = 6). ** for *P* < 0.01 vs. WT.

We previously reported that inactivation of (p)ppGpp signaling leads to impaired SOD expression and activity, and that SODs confer multidrug tolerance in stationary phase *P. aeruginosa* (Martins et al., 2018). *P. aeruginosa* encodes two different SODs, SodA and SodB. The Fe-cofactored SodB is most abundant in iron replete conditions, while the Mn-cofactored SodA is only expressed under iron limitation or in the absence of SodB (Hassett et al., 1995, 1996). Since the *sodB* mutant retains 10–15% of the SOD activity of WT cells in stationary phase (Hassett et al., 1995; Martins et al., 2018), we proceeded to characterize the *sodAB* mutant where both *sodA* and *sodB* genes are inactivated (Iiyama et al., 2007). As expected, the *sodAB* mutant exhibited no detectable SOD activity (Figure 1A) and elevated intracellular superoxide levels as indicated from its DHE/EtBr fluorescence ratio (Figure 1B). Furthermore, stationary phase *sodAB* cells display ∼3-log_10_ greater killing by ofloxacin (Figure 1C) and meropenem (Figure 1D) than the WT strain. No difference in antibiotic killing was observed between these two strains during the exponential growth phase (Supplementary Figure S1), which further supports our recent finding that SODs are required specifically for stationary phase antibiotic tolerance (Martins et al., 2018).

### Superoxide Generators Induce Antibiotic Tolerance

**FIGURE 2:**
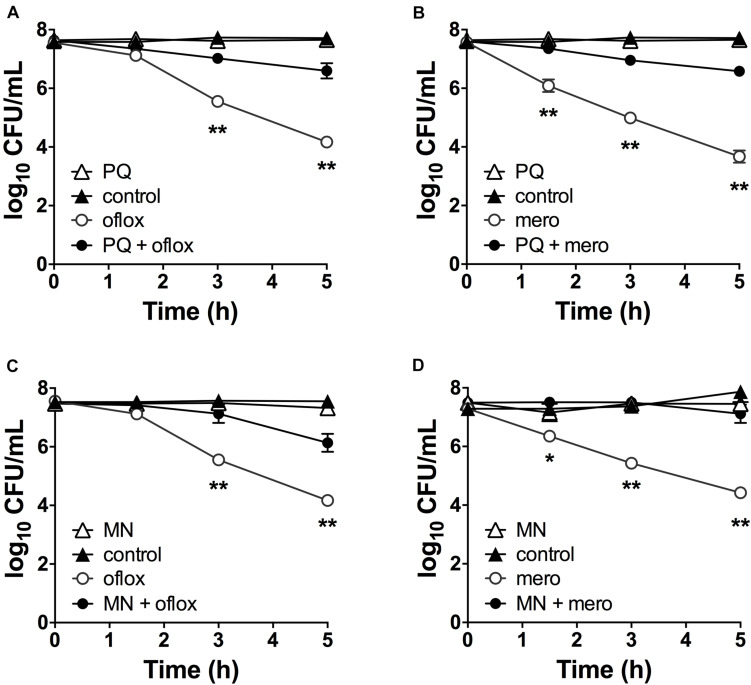
Sublethal pre-challenge with PQ and MN enhance drug tolerance. Killing assays for stationary phase WT cells with **(A,C)** ofloxacin 5 μg/mL and **(B,D)** 500 μg/mL meropenem ± pre-challenge with **(A,B)** 1.25 mM PQ or **(C,D)** 0.175 mM MN for 20 min before addition of antibiotic. Note that the data points for PQ alone and vehicle controls overlap **(A,B)**. Results are shown as mean ± SEM (*n* = 6). * for *P* < 0.05 and ** for *P* < 0.01 vs. antibiotic treatment alone.

Although superoxide-generating compounds can cause cell toxicity and death, we hypothesized that at sublethal doses they induce adaptive responses that confer protection against antibiotics. To examine this possibility, we selected paraquat (PQ) and menadione (MN), two well-known chemically distinct and cell-permeable superoxide generators. Prior to antibiotic challenge, wild-type (WT) cells were pre-incubated with 1.25 mM PQ or 0.175 mM MN at sublethal concentrations that did not affect bacterial viability (Figures 2A,B). While ofloxacin and meropenem alone caused bacterial killing with 2.5- to 3.0-log_10_ lower viable counts compared to control conditions, pre-incubation with PQ nearly completely abrogated ofloxacin (Figure 2A) and meropenem (Figure 2B) killing of WT cells. Similar results were observed with sublethal 0.175 mM MN and ofloxacin (Figure 2C) or meropenem (Figure 2D) killing.

### PQ and MN Rapidly Induce SOD Activity and This Requires *De Novo* Protein Synthesis

**FIGURE 3:**
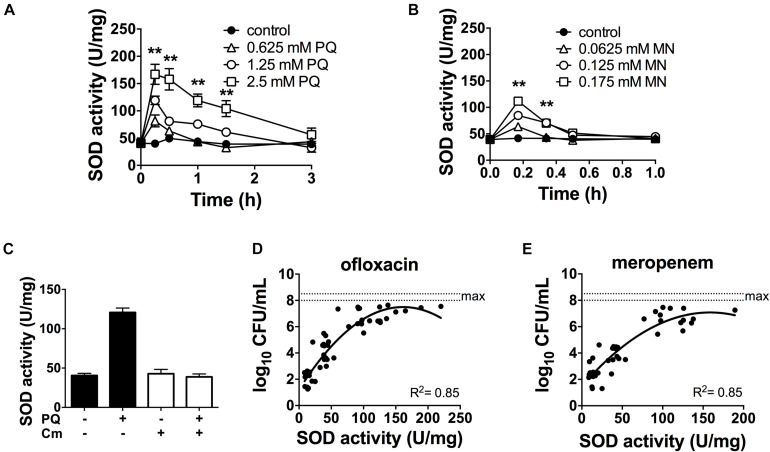
SOD activity is induced by PQ and MN, requires *de novo* protein synthesis and is a determinant of antibiotic tolerance. Dose- and time-response of SOD activity following challenge of stationary phase WT cells with sublethal **(A)** PQ, **(B)** MN or vehicle (control). **(C)** Stationary phase WT cells was treated with 500 μg/mL chloramphenicol (Cm) for 1 h to inhibit *de novo* protein synthesis, then challenged with 1.25 mM PQ for 20 min before SOD activity was measured. Correlation between SOD activity and **(D)** ofloxacin and **(E)** meropenem tolerance was established by simultaneously measuring in the same sample the SOD activity and the bacterial survival following a 5 h challenge with 5 μg/mL ofloxacin or 500 μg/mL meropenem. The max counts (dotted lines) were defined by bacterial counts in untreated controls. Combined data from different strains (WT, Δ*relA spoT*, *rpoS*, and Δ*soxR*) in conditions with 1.25 mM PQ, 0.175 mM MN or vector controls are shown and each data point represents one independent replicate (*n* ≥ 25). The correlation coefficient R^2^ was calculated using non-linear (second order polynomial) regression. For **(A–C)**, results are shown as mean ± SEM (*n* = 6) and ** for *P* < 0.01 vs. untreated WT.

Since antibiotic tolerance is impaired upon loss of SODs (Figures 1C,D), and high SOD activity achieved through genetic or chemical complementation confers antibiotic tolerance (Martins et al., 2018), we reasoned that PQ might confer tolerance by inducing SOD activity. As shown in Figure 3A, sublethal concentrations of PQ (0.625–2.5 mM) rapidly induce SOD activity by 1.5- to 4.0-fold in a dose dependent manner. Activity levels reach a maximum within 15 min and return to baseline levels over 1–3 h. We note that, in order to generate a robust SOD induction at low PQ concentrations, stationary phase cultures were diluted in their own spent supernatant to reduce the cell concentration without providing new nutrients or stimulating growth. As a control, we confirmed that diluted and undiluted cells showed a comparable dose-dependent SOD induction in response to PQ (Supplementary Figure S2) as well as PQ-induced ofloxacin tolerance (Supplementary Figure S3). Finally, we also tested MN, a chemically distinct superoxide generator and found that it had similar effects, with a 3-fold induction of SOD activity by 0.175 mM MN within 20-min of challenge (Figure 3B).

The rapid SOD response following PQ challenge led us to ask if it required *de novo* protein synthesis. To test this, we inhibited protein synthesis with the bacteriostatic antibiotic chloramphenicol. We first validated this approach using an arabinose-inducible *katA* expressing construct (pBAD*-katA*), measured catalase activity in the presence or absence of 500 μg/mL chloramphenicol, and confirmed that pre-treatment with chloramphenicol abrogates the arabinose-dependent induction of catalase activity (Supplementary Figures S4A,B). Next, we demonstrated that PQ-mediated induction of SOD activity is completely abolished by pre-treatment with chloramphenicol (Figure 3C). Whether the induction of SOD activity requires *de novo* synthesis of SOD itself, or indirectly via another protein remains to be determined. Finally, we noted that a strong correlation between SOD activity and antibiotic tolerance in PQ- and MN-treated cells (Figures 3D,E) is comparable to untreated cells from our previous report (Martins et al., 2018).

### PQ-Induced Tolerance Is SOD Dependent and Can Rescue the Δ*relA spoT* Mutant

**FIGURE 4:**
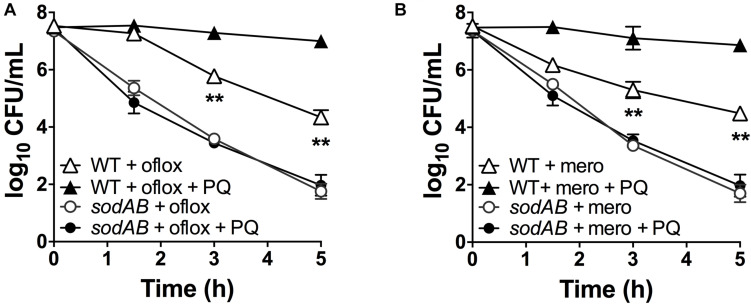
SOD deletion abrogates PQ-induced tolerance. Stationary phase WT or *sodAB* mutant cells were pre-challenged with 1.25 mM PQ and assayed 20 min later for killing with **(A)** 5 μg/mL ofloxacin or **(B)** 500 μg/mL meropenem. Results are shown as mean ± SEM (*n* = 6). ** for *P* < 0.01 vs. the corresponding strains treated with antibiotic alone.

If stimulation of SOD activity is responsible for PQ-mediated antibiotic tolerance, we reasoned that this effect would be abrogated in the *sodAB* mutant. We thus treated this mutant with 1.25 mM PQ prior to challenge with ofloxacin and meropenem. First, we confirmed that sublethal PQ did not cause any loss of viability in WT or *sodAB* cells under our experimental conditions (Supplementary Figure S5). Next, we observed that ofloxacin (Figure 4A) and meropenem (Figure 4B) killing of the *sodAB* mutant was identical in the presence or absence of PQ, demonstrating that PQ-induced tolerance requires *sodA* and/or *sodB*.

**FIGURE 5:**
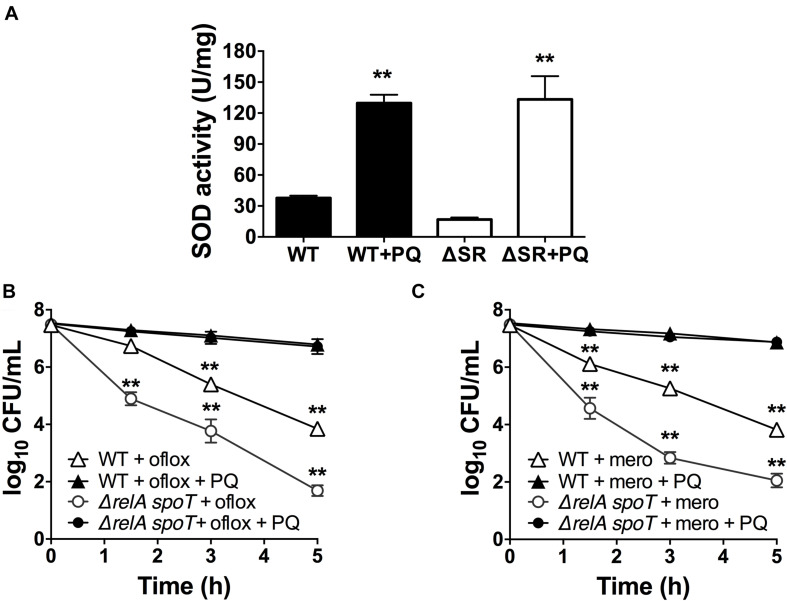
PQ induces SOD activity and antibiotic tolerance in the Δ*relA spoT* mutant. Stationary phase WT or the (p)ppGpp-null Δ*relA spoT* cells were pre-challenged with 1.25 mM PQ and assayed 20 min later for **(A)** SOD activity and killing with **(B)** 5 μg/mL ofloxacin or **(C)** 500 μg/mL meropenem. Results are shown as mean ± SEM (*n* = 6). ** for *P* < 0.01 vs. untreated controls in **(A)** and the respective strains treated with antibiotic alone in **(B,C)**.

Since loss of (p)ppGpp signaling in the Δ*relA spoT* mutant causes a SOD defect and impaired stationary phase antibiotic tolerance, and we previously demonstrated that genetic and chemical SOD complementation rescued the tolerance of the Δ*relA spoT* mutant (Nguyen et al., 2011; Martins et al., 2018), we asked whether pre-challenge with sublethal PQ was also sufficient to restore antibiotic tolerance. We first confirmed that the Δ*relA spoT* mutant displays ∼3-fold lower SOD activity compared to WT (Figure 5A), and 2- to 3-log_10_ greater killing by ofloxacin (Figure 5B) and meropenem (Figure 5C). Then, we demonstrated that sublethal PQ does not alter the viability of the Δ*relA spoT* mutant (Supplementary Figure S3C) but increases its SOD activity by 8-fold to levels comparable to those in PQ-treated WT cells (Figure 5A). Hence, under our conditions, PQ restored the tolerance of the Δ*relA spoT* mutant to WT levels, with a reduction of 5- and 4.8-log_10_ in killing by ofloxacin (Figure 5B) and meropenem (Figure 5C), respectively. This indicates that the PQ-induced SOD response does not require (p)ppGpp and is sufficient to restore drug tolerance to the Δ*relA spoT* mutant.

### PQ-Induced Antibiotic Tolerance in *P. aeruginosa* Requires RpoS but Not SoxR

**FIGURE 6:**
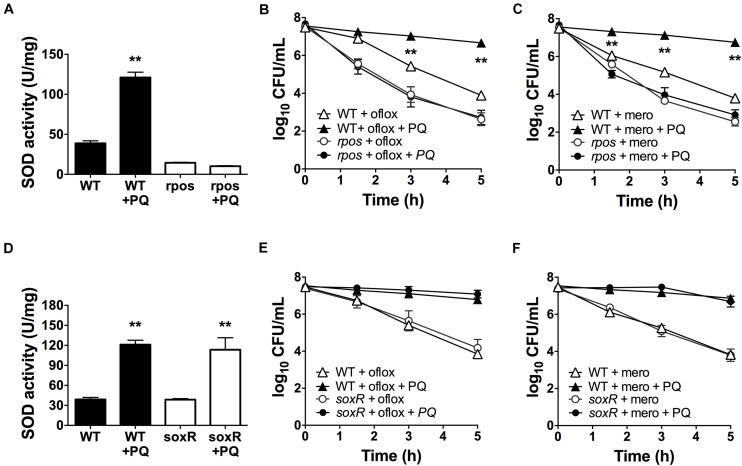
PQ-induced SOD activity and multidrug tolerance requires RpoS but not SoxR. Stationary phase WT, *rpoS* or Δ*soxR* cells were pre-challenged with 1.25 mM PQ and assayed 20 min later for **(A,D)** SOD activity and killing with **(B,E)** 5 μg/mL ofloxacin or **(C,F)** 500 μg/mL meropenem. Note that the data point for *rpoS* with and without PQ overlap in **(B,C)**. Results are shown as mean ± SEM (*n* = 6). ** for *P* < 0.01 vs. untreated controls (for **A,D**) and WT cells challenged with the same treatment (for **B,C,E,F**).

The alternative sigma factor RpoS regulates SOD expression (Martins et al., 2018) and the transcriptional factor SoxR is activated by PQ (Greenberg et al., 1990; Kobayashi and Tagawa, 2004). Thus, we sought to determine whether RpoS and SoxR were involved in the PQ-induced SOD response. First, we tested the *rpoS* mutant and found that, in contrast to WT cells, sublethal PQ does not induce any SOD activity (Figure 6A) or antibiotic tolerance to ofloxacin (Figure 6B) or meropenem (Figure 6C). In contrast, PQ induces SOD activity (Figure 6D) and drug tolerance (Figures 6E,F) to the same extent in the *soxR* mutant as in WT cells. As controls, we also confirmed that PQ does not affect the viability of either the *soxR* or *rpoS* mutant (Supplementary Figures S3B,D). These results therefore indicate that the PQ-induced responses in stationary phase *P. aeruginosa* require RpoS but not the PQ-responsive SoxR.

### PQ Lowers Envelope Permeability and Ofloxacin Internalization

**FIGURE 7:**
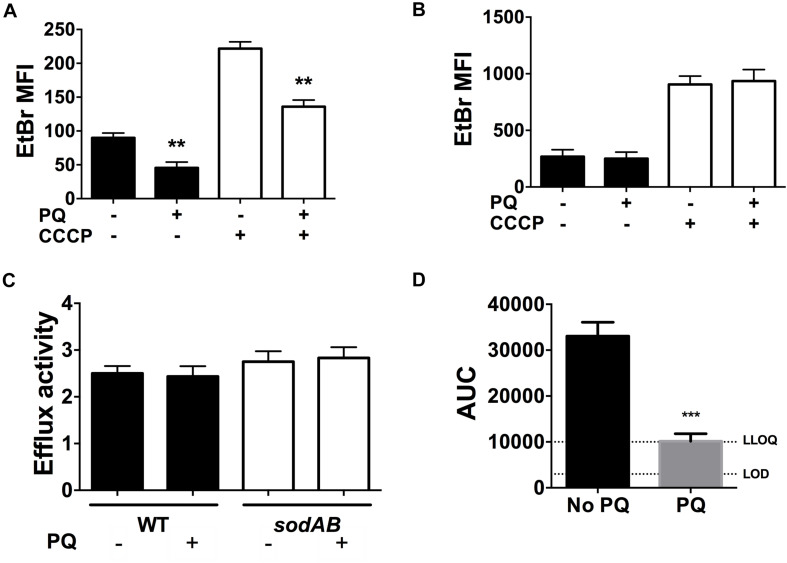
PQ lowers envelope permeability and ofloxacin internalization in a SOD-dependent but efflux independent fashion. EtBr internalization was measured in **(A)** WT and **(B)** *sodAB* stationary phase cells ±1.25 mM PQ pre-challenge and ±100 μM CCCP. **(C)** Relative efflux activity was estimated by the ratio of EtBr mean fluorescence intensity (MFI) in (+)CCCP to (-)CCCP conditions. **(D)** Ofloxacin internalization over 1 h was measured by LC/MS in the WT strain ± pre-challenge with 1.25 mM PQ for 20 min. Relative ofloxacin concentrations are reported as area under the curve (AUC), with the lower limit of detection (LOD) and lower limit of quantification (LLOQ) indicated. All results are shown as mean ± SEM (*n* ≥ 6). ** for *P* < 0.01 or *** for *P* < 0.001 vs. the same condition without PQ.

We recently reported that SODs lower envelope permeability and restrict drug internalization in stationary phase cells (Martins et al., 2018). We thus examined the effect of PQ on envelope permeability by measuring EtBr internalization as a relative measure of envelope permeability. As shown in Figure 7A, EtBr internalization in WT cells is diminished by 2.5-fold after PQ exposure. Since EtBr internalization is a function of both envelope permeability and H^+^-dependent efflux activity, we also measured EtBr internalization in the presence of the ionophore CCCP to inactivate efflux pumps. As expected, CCCP increases intracellular EtBr fluorescence and this effect was similar for cells pre-challenged with PQ (Figure 7A).

To probe if PQ-induced reduction in envelope permeability is SOD-dependent, we measured EtBr internalization in the *sodAB* mutant in the presence or absence of PQ. PQ has no effect on EtBr internalization in this mutant (Figure 7B), in contrast to WT cells (Figure 7A), indicating that PQ’s effect on envelope permeability also required SOD activity. We also noted that *sodAB* mutant cells exhibit significantly higher EtBr fluorescence compared to WT cells whether CCCP is present or absent (Figure 7C). Moreover, the ratio of EtBr fluorescence with and without CCCP, i.e., (+) CCCP/(-) CCCP, an indicator of relative efflux activity, are similar between the WT and *sodAB* mutant and are not affected by PQ. Together, these results suggest that PQ lowers envelope permeability in an efflux-independent fashion.

Finally, we examined the effect of PQ on the accumulation of ofloxacin in stationary phase WT cells. As shown in Figure 7D, PQ-treated WT cells internalize ∼3.3-fold less ofloxacin than untreated cells, which we attribute to PQ-mediated reduction in envelope permeability.

## Discussion

This study expanded on our recent work and further confirmed a key role for SOD activity in mediating multidrug tolerance in stationary phase *P. aeruginosa* (Martins et al., 2018). We here report that deletion of SOD activity in *sodAB* cells increases intracellular superoxide levels and antibiotic killing. Pre-challenge with sublethal paraquat (PQ) and menadione (MN) nearly abolishes antibiotic killing of WT cells but this rescue is completely abrogated in the *sodAB* mutant, indicating that it is SOD-dependent. We determined that RpoS is required PQ-induced increase in SOD activity and antibiotic tolerance, but not SoxR or (p)ppGpp signaling. We previously reported that SODs are positively regulated by (p)ppGpp signaling (Nguyen et al., 2011; Martins et al., 2018) and RpoS (Murakami et al., 2005) under basal growth conditions, and we now found that only RpoS is required for SOD induction under PQ challenge. How RpoS upregulates SOD activity in PQ-challenged *P. aeruginosa* remains to be determined. We also found no evidence that PQ increased H^+^-dependent efflux activity in *P. aeruginosa*. In fact, PQ lowers envelope permeability in a SOD-dependent but efflux-independent fashion. Our current and recently published results show that the SOD-dependent reduction in envelope permeability is associated with a concurrent reduction in internalization of ofloxacin, as well as meropenem (Martins et al., 2018). In the absence of altered drug efflux, this most likely indicates a reduction in drug penetration, although other mechanisms such as drug degradation cannot be excluded. How SOD activity alters envelope permeability and drug penetration remain to be elucidated.

PQ and MN are redox-cycling drugs that generate superoxide radicals, that can directly damage [2Fe-2S] clusters and oxidize NADPH-reduced enzymes, leading to inactivation of dehydratases and NADPH depletion (Gu and Imlay, 2011). PQ induces the expression of several anti-oxidant defenses, including the *katB* catalase and alkyl hydroperoxidases *ahpBCF* through an OxyR-dependent response (Ochsner et al., 2000; Hare et al., 2011) as well as *sodA* and *sodB* gene expression, leading to increased SOD activity levels in *E. coli* (Pomposiello et al., 2001; Chen et al., 2006; Hare et al., 2011), and *P. aeruginosa* (Hare et al., 2011). In *E. coli*, induction of SodA upon PQ challenge is SoxR dependent (Pomposiello et al., 2001), and RpoS positively regulates *sodA* expression (Wong et al., 2017). PQ directly activates SoxR through oxidation of its [2Fe-2S] cluster (Gu and Imlay, 2011), which in *E. coli*, leads to the expression of the transcriptional regulator SoxS, which in turn regulates >100 genes (Pomposiello et al., 2001). In addition to genes involved in superoxide detoxification such as *sodA* (Greenberg et al., 1990), the *E. coli* SoxRS regulon includes genes involved in efflux systems (MarR-AB, AcrAB-TolC) that extrude antibiotics (Pomposiello et al., 2001; Wu et al., 2012), membrane porins (MicF and OmpF) implicated in drug influx (Chou et al., 1993), and LPS modification (*waaY*) (Lee J.H. et al., 2009).

PQ activation of the SoxRS system has been previously linked to antibiotic resistance in *E. coli* (Miller et al., 1994; Koutsolioutsou et al., 2001, 2005). Miller et al. (1994) reported that PQ dampens the antibacterial activity of enoxacin, a fluoroquinolone, and that this effect requires the superoxide-responsive SoxRS system. Wu et al. (2012) reported that PQ increased fluoroquinolone resistance in *E. coli*, an effect abrogated in the *acrB* mutant, suggesting that it may be mediated by the AcrAB-TolC drug efflux system. Interestingly, Mosel et al. (2013) also observed that PQ induced MarA and AcrAB-TolC but deletion of these efflux systems was not sufficient to abrogate the PQ-mediated tolerance to oxolinic acid, kanamycin and ampicillin, indicating that mechanisms other than these efflux systems were involved. Notably, none of these *E. coli* studies specifically measured envelope permeability nor drug susceptibility in stationary phase cells, and it is uncertain whether the PQ-mediated tolerance observed in our studies shares common mechanisms with the above *E. coli* studies.

Although *P. aeruginosa* possesses the superoxide-sensing SoxR, it lacks SoxS (Kobayashi and Tagawa, 2004). Previous studies have also highlighted the divergent roles of SoxR in *E. coli* and *P. aeruginosa* (Palma et al., 2005; Dietrich et al., 2008; Singh et al., 2013). Its six gene SoxR regulon includes the *mexGHI-ompD* efflux system and PA3718, a putative efflux pump, but not *sodA* nor *sodB* (Palma et al., 2005). Our demonstration that *soxR* deletion has no impact on PQ-induced SOD activity nor antibiotic tolerance thus implies that SoxR-regulated efflux systems do not explain PQ-induced antibiotic tolerance. Furthermore, we did not detect significant changes in efflux activity following PQ treatment, thus suggesting that drug efflux is unlikely to be a major mechanism of PQ-induced tolerance.

We recognize that redox-cycling agents such as PQ have pleiotropic effects on gene expression and protein activity, some of which may contribute to the inducible tolerance observed in our conditions. PQ modulates gene expression through SoxR, SoxR-independent responses including OxyR and Fur, or indirectly through perturbation of redox enzymes (Ochsner et al., 2000; Blanchard et al., 2007). In *P. aeruginosa*, GeneChip experiments by Salunkhe et al. looking at the global gene expression of stationary phase bacteria in response to 0.5 mM PQ only identified 0.5% of ORFs to be differentially expressed. In the *P. aeruginosa* PAO1 strain, PQ upregulated genes involved in the TCA cycle and acetoin metabolism (e.g., acetyl-coenzyme A synthase *acsA*, acetoin catabolism *acoB*), in membrane transport (a putative sodium/solute symporter PA3234, the ABC transporter PA4502-4506), the *opdQ* and *oprC* outer membrane porins, and *fpr* encoding the ferredoxin NADP reductase, while six genes were down-regulated, including PA0105-0108 encoding the cytochrome c oxidase subunits (Salunkhe et al., 2002). PQ and other redox-cycling drugs can also directly oxidize and inactivate catalytic [2Fe-2S] clusters of dehydratases involved in carbon and energy metabolism, leading to disruption in metabolic pathways and respiration (Kuo et al., 1987; Gu and Imlay, 2011). Multiple groups have reported that fluctuations in ATP levels and cellular respiration, central carbon metabolism, and expression of energy generating components are linked to persister formation and antibiotic tolerance (Amato et al., 2014; Lobritz et al., 2015; Orman and Brynildsen, 2015; Meylan et al., 2017; Zalis et al., 2019). It is therefore possible that PQ triggers alterations in central carbon and energy metabolism, which also contribute to ofloxacin and meropenem tolerance in our conditions. We note however that our experiments challenged stationary phase cells with PQ at sublethal concentrations, and the effects such concentrations have on carbon and energy metabolism remain to be determined. Further studies would be required to evaluate these specific mechanisms.

The present observation that SODs mediate the PQ-induced antibiotic tolerance is consistent with our recent report that genetic complementation with *sodA* and *sodB*, and chemical complementation with the SOD mimetic Mn(III)-tetrakis-(1-methyl-4-pyridyl) porphyrin pentachloride were also sufficient to restore antibiotic tolerance to the Δ*relA spoT* mutant (Martins et al., 2018). These results thus further support the important contribution of SOD activity to antibiotic tolerance, which is growth phase specific and negligible under rapid growth conditions as the absence of SOD activity does not affect antibiotic tolerance in exponentially growing *P. aeruginosa*. Our results with the *sodAB* mutant are consistent with studies in *Enterococcus faecalis* (Bizzini et al., 2009; Ladjouzi et al., 2015), *Campylobacter jejuni* (Hwang et al., 2013), *Acinetobacter baumannii* (Heindorf et al., 2014), and *E. coli* (Dwyer et al., 2007; Wang et al., 2014), where loss of SODs also enhances bactericidal antibiotic killing. These stand in contrast to other studies performed in exponentially growing *E. coli* reporting that the *sodA sodB* mutant exhibited similar susceptibility to ampicillin, gentamicin, and norfloxacin killing to the wild-type strain (Wang and Zhao, 2009; Ezraty et al., 2013), and that overexpression of *sodA* or *sodB* did not mitigate ampicillin and ofloxacin killing (Orman and Brynildsen, 2016). Why SOD activity is critical to antibiotic survival during stationary phase but not exponential phase remains to be determined. Dukan and Nystrom previously reported that SOD-deficient *E. coli* mutants exhibit increased protein oxidation only in stationary phase cultures (Dukan and Nystrom, 1999) and proposed that superoxide stress was a hallmark of respiring but non-replicating stationary phase cells (Dukan and Nystrom, 1998).

Several groups have linked antibiotic lethality with ROS mediated toxicity (Dwyer et al., 2007; Grant et al., 2012; Hwang et al., 2013). Hence, reports that PQ and other redox-cycling compounds that generate superoxide radicals actually mitigate antibiotic killing (Wu et al., 2012; Mosel et al., 2013), while superoxide generating nanoparticles (Courtney et al., 2017) and plumbagin enhance isoniazid toxicity in *Mycobacterium tuberculosis* (Bulatovic et al., 2002) and *Mycobacterium smegmatis* (Wang et al., 1998), raised questions about the paradoxical role of superoxide stress and SOD in conferring protection and toxicity, respectively, upon antibiotic challenge. Based on our current study, we propose that PQ-induced tolerance has the hallmark of hormesis, a phenomenon in which low doses of a stressor induce a response protective against subsequent high doses of the same or different stressor (Calabrese and Mattson, 2017). In other words, pre-challenge of cells with sublethal PQ or MN induces a SOD response that protects them against an ensuing antibiotic stress. Thus, whether superoxide confers protection or enhances antibiotic lethality will depend on dosage and growth phase. Furthermore, the notion of hormesis and stress-induced tolerance may be relevant beyond sublethal PQ stress, as other physiological cues modulate SOD responses. During *in vivo* infections where bacteria encounter nutrient limitations, host-derived ROS and other challenges, multiple stress-induced responses may dampen the lethality of antibiotics. For example, Rowe et al. (2020) recently reported that ROS generation within macrophage phagolysosome induced multidrug tolerance in *S. aureus*. Chemical or metabolic perturbations aimed at potentiating antibiotic lethality should thus be evaluated in physiological contexts relevant to bacteria growing *in vivo*.

## Data Availability Statement

The original contributions presented in the study are included in the article/Supplementary Material, further inquiries can be directed to the corresponding author/s.

## Author Contributions

DM and GM generated and analyzed the data. DM, AME, and DN designed the study and wrote the manuscript. All authors contributed to the article and approved the submitted version.

## Conflict of Interest

The authors declare that the research was conducted in the absence of any commercial or financial relationships that could be construed as a potential conflict of interest.

## Supplementary Material

FIGURE S1 Loss of SOD activity does not affect drug tolerance in exponential phase *P. aeruginosa.* Wild-type (WT) and *sodAB* cells were grown to OD_600_ = 0.2 and challenged with **(A)** 5 μg/mL ofloxacin and **(B)** 500 μg/mL meropenem. Note that the data points for WT and *sodAB* without antibiotics overlap in **(A,B)**. Results are shown as mean ± SD (*n* = 3).

FIGURE S2 PQ induction of SOD activity is concentration dependent in both undiluted and diluted cultures of stationary phase *P. aeruginosa.* Stationary phase WT cells were **(A)** undiluted or **(B)** diluted 10-fold in their own culture supernatant prior to challenge with different sublethal concentrations of PQ for 1.5 h, followed by measurement of SOD activity. Results are shown as mean ± SD (*n* = 3). ^∗^ for *P* < 0.05 and ^∗∗^ for *P* < 0.01 vs. untreated controls.

FIGURE S3 Pre-challenge with sublethal PQ confer ofloxacin tolerance to both undiluted and diluted cultures of stationary phase *P. aeruginosa.* Killing assays with ofloxacin 5 μg/mL in **(A)** undiluted stationary phase WT cells ± pre-challenge with 7.5 mM PQ or **(B)** 10-fold diluted stationary phase WT cells with 1.25 mM PQ for 1.5 h before addition of antibiotic. Note that the data points for PQ alone and vehicle controls overlap **(A,B)**. Representative plots are shown as mean ± SD (*n* = 3). ^∗∗^ for *P* < 0.01 vs. antibiotic treatment alone.

FIGURE S4 Chloramphenicol inhibits *de novo* catalase activity of WT expressing *pBAD-katA*. Catalase activity in stationary phase WT cells expressing the **(A)** *pBAD* vector control or **(B)** arabinose inducible catalase construct *pBAD-katA.* Cells were incubated ±2% wt/v arabinose (Ara) and ± 500 μg/mL chloramphenicol (Cm) for 1.25 h at 37°C with shaking at 250 rpm. Results are shown as mean ± SEM (*n* ≥ 6). ^∗∗^ for *P* < 0.01 vs. the untreated control (-Ara, -Cm).

FIGURE S5 Challenge with 1.25 mM PQ does not affect bacterial viability. Bacterial viability of stationary phase **(A)** *sodAB*, **(B)** *soxR*, **(C)** Δ*relA spot*, and **(D)** *rpoS* mutant cells challenged with 1.25 mM PQ over 5 h at 37°C with shaking at 250 rpm. Results are shown as mean ± SEM (*n* = 6).

FIGURE S6 Bacterial viability remains unchanged during ofloxacin internalization assay. Stationary phase WT cells were pre-challenged with or without 1.25 mM PQ for 20 min, then incubated with 0.5 μg/mL ofloxacin for 1 h. Bacterial viability was measured by CFU count in samples prior to and after ofloxacin incubation for the internalization assay. Results are shown as mean ± SEM (*n* = 6).
