# Supplementary material for: Sublethal Paraquat Confers Multidrug Tolerance in Pseudomonas aeruginosa by Inducing Superoxide Dismutase Activity and Lowering Envelope Permeability
Source: Front Microbiol. 2020 Sep 25;11:576708. doi: 10.3389/fmicb.2020.576708 (PMC7546422; doi:10.3389/fmicb.2020.576708)
Supplement: FIGURE S1 — Loss of SOD activity does not affect drug tolerance in exponential phase P. aeruginosa. Wild-type (WT) and sodAB cells were grown to OD600 = 0.2 and challenged with (A) 5 μg/mL ofloxacin and (B) 500 μg/mL meropenem. Note that the data points for WT and sodAB without antibiotics overlap in (A,B). Results are shown as mean ± SD (n = 3). [file Data_Sheet_1.pdf]

Supplemental figures

Sublethal paraquat confers multidrug tolerance in *Pseudomonas aeruginosa* by inducing superoxide dismutase activity and lowering envelope permeability

Running title: Paraquat-induced antibiotic tolerance in *P. aeruginosa*

Dorival Martins[1,2], Geoffrey A. McKay[1], Ann M. English[4], Dao Nguyen[1,2,3,#]

[1] Meakins-Christie Laboratories, Research Institute of the McGill University Health Centre
[2] Department of Microbiology and Immunology McGill University
[3] Department of Medicine, McGill University
[4] Department of Chemistry and Biochemistry, Concordia University

[#]Corresponding author. dao.nguyen@mcgill.ca

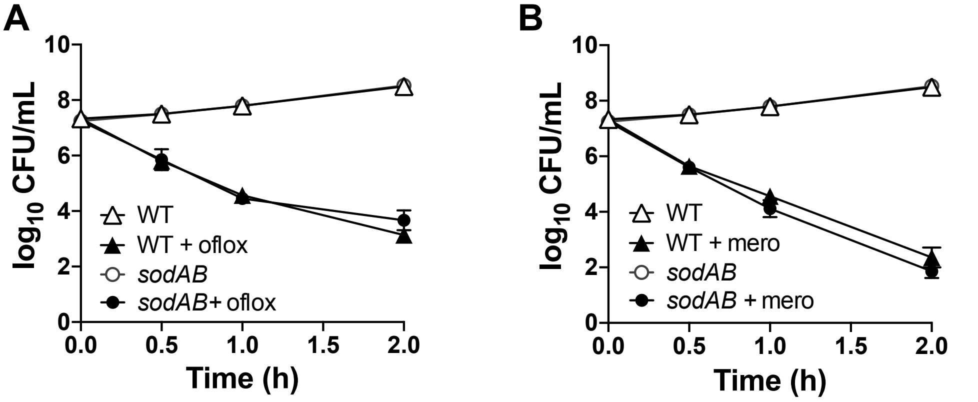

Suppl. Fig. S1: Loss of SOD activity does not affect drug tolerance in exponential phase *P. aeruginosa.* Wild-type (WT) and *sodAB* cells were grown to OD_{600}= 0.2 and challenged with (A) 5 µg/mL ofloxacin and (B) 500 µg/mL meropenem. Note that the data points for WT and *sodAB* without antibiotics overlap in (A) and (B). Results are shown as mean ± SEM (n=3).

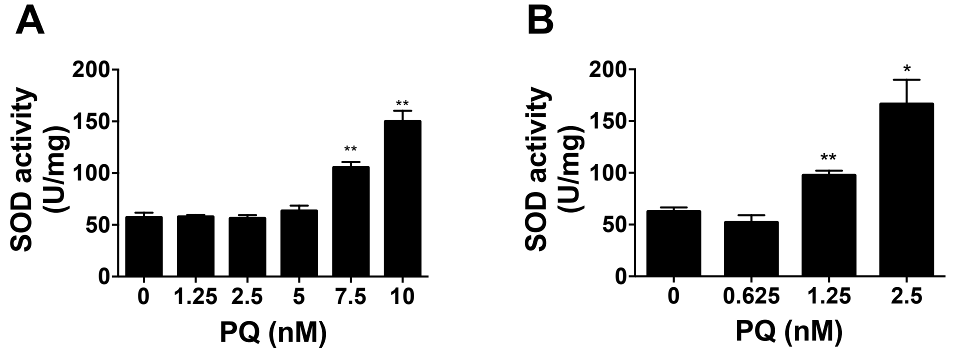

Suppl. Fig. S2: PQ induction of SOD activity is concentration dependent in both undiluted and diluted cultures of stationary phase *P. aeruginosa.* Stationary phase WT cells were (A) undiluted or (B) diluted 10-fold in their own culture supernatant prior to challenge with different sublethal concentrations of PQ for 1.5h, followed by measurement of SOD activity. Results are shown as mean ± SD (n=3). * for P< 0.05 and ** for P< 0.01 vs. untreated controls.

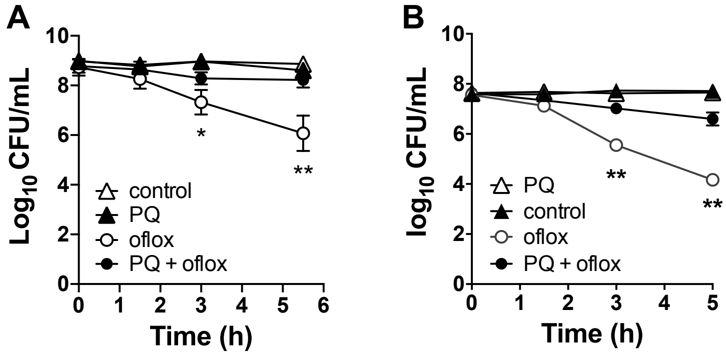

Suppl. Fig. S3: Pre-challenge with sublethal PQ confer ofloxacin tolerance to both undiluted and diluted cultures of stationary phase *P. aeruginosa.* Killing assays with ofloxacin 5 µg/mL in (A) undiluted stationary phase WT cells ± pre-challenge with 7.5 mM PQ or (B) 10-fold diluted stationary phase WT cells with 1.25 mM PQ for 1.5 h before addition of antibiotic. Note that the data points for PQ alone and vehicle controls overlap (A) and (B). Representative plots are shown as mean ± SD (n=3). ** for P< 0.01 vs. antibiotic treatment alone.

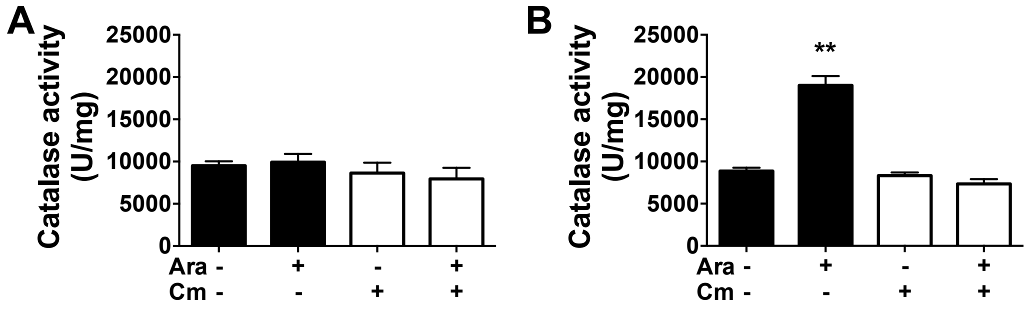

Suppl. Fig. S4: Chloramphenicol inhibits *de novo* catalase activity of WT expressing *pBAD-katA*. Catalase activity in stationary phase WT cells expressing the (A) *pBAD* vector control or (B) arabinose inducible catalase construct *pBAD-katA*. Cells were incubated ±2% wt/v arabinose (Ara) and ±500 µg/mL chloramphenicol (Cm) for 1.25 h at 37°C with shaking at 250 r.p.m. Results are shown as mean ± SEM (n≥6). ** for P< 0.01 vs. the untreated control (-Ara, -Cm).

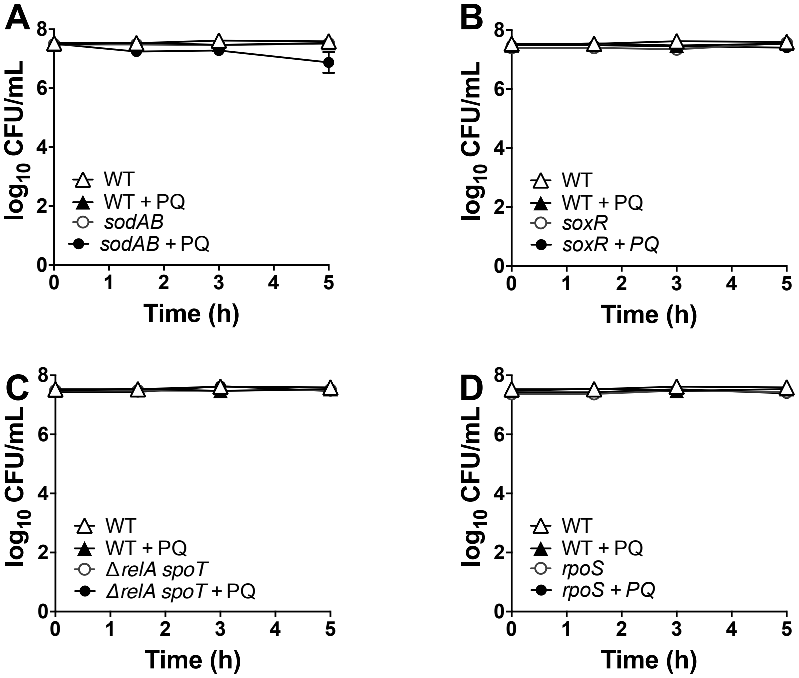

Suppl. Fig. S5: Challenge with 1.25 mM PQ does not affect bacterial viability. Bacterial viability of stationary phase (A) *sodAB,* (B) *soxR,* (C) Δ*relA spoT* and (D) *rpoS* mutant cells challenged with 1.25 mM PQ over 5 h at 37°C with shaking at 250 r.p.m. Results are shown as mean ± SEM (n=6).

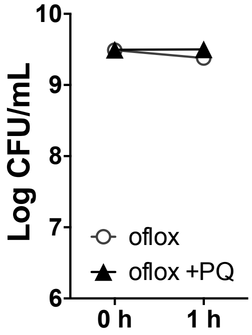

Suppl. Fig. S6: Bacterial viability remains unchanged during ofloxacin internalization assay. Stationary phase WT cells were pre-challenged with or without 1.25 mM PQ for 20 min, then incubated with 0.5 µg/mL ofloxacin for 1h. Bacterial viability was measured by CFU count in samples prior to and after ofloxacin incubation for the internalization assay. Results are shown as mean ± SEM (n=6).
